# Correlation between T-Wave Alternans and Cardiac Volume Status via Intrathoracic Impedance Measurements

**DOI:** 10.1155/2012/167562

**Published:** 2012-09-04

**Authors:** Jose' Dizon, Kathleen Hickey, Hasan Garan

**Affiliations:** ^1^Division of Cardiology, Department of Medicine and Columbia University Medical Center, Columbia University, New York, NY 10032, USA; ^2^222 Westchester Avenue, White Plains, NY 10604, USA

## Abstract

*Introduction*. The presence of T-wave alternans (TWA) has been shown to correlate with a higher risk for sudden cardiac death. The mechanism of TWA may be related to abnormalities in intracellular calcium handling, which is a mechanism in heart failure and associated arrhythmias as well. However, an association between TWA and cardiac volume status has not been demonstrated. *Methods Used*. We report the case of a 54-year-old man with a dilated cardiomyopathy who had a biventricular defibrillator system implanted with intrathoracic impedance measurement capability. We performed baseline TWA testing, which was normal and was associated with normal clinical status and normal intrathoracic impedance. We followed intrathoracic impedance measurements, and when the measurement suggested volume overload eight months later, we repeated the TWA test. TWA was grossly positive, and volume overload was corroborated with clinical heart failure. The patient was diuresed, and when clinical status and intrathoracic impedance returned to normal a month later, we repeated TWA, which was again negative. *Conclusion*. This case demonstrates a correlation between cardiac volume status, as measured by intrathoracic impedance measurements, and TWA status. This data suggests that conditions of volume overload such as heart failure could be causally related to increased TWA, perhaps by the common mechanism of altered intracellular calcium handling.

## 1. Introduction

The presence of microvolt T-wave alternans has been shown to correlate with a higher risk of sudden cardiac death in patients with structural heart disease [1]. The mechanism of T-wave alternans is unclear, but may be related to altered intracellular calcium handling, also commonly observed in congestive heart failure [2, 3]. Altered calcium cycling and mechanical stretch in heart failure have been implicated in the development of repolarization abnormalities and arrhythmogenesis [4, 5]. Certain models of implantable defibrillators have the capability of monitoring cardiac fluid status via intrathoracic impedance measurements. We sought to determine if a correlation exists between T-wave alternans and cardiac volume status, as monitored by intrathoracic impedance (Optivol, Medtronic, Inc., Minneapolis, MN, USA). Patients with implanted biventricular defibrillator systems for dilated cardiomyopathy, Class III congestive heart failure, and left bundle branch block had T-wave alternans testing via the spectral method (Cambridge Heart, Inc.) by atrial pacing up to 110 bpm during routine defibrillator followup (or atrial-biventricular pacing in cases of atrioventricular block). Intrathoracic impedance measurements were performed monthly by home monitoring of the defibrillator system. When impedance measurements suggested fluid overload, the patients returned to the clinic for T-wave alternans testing.

## 2. Case Report

**Figure 1:**
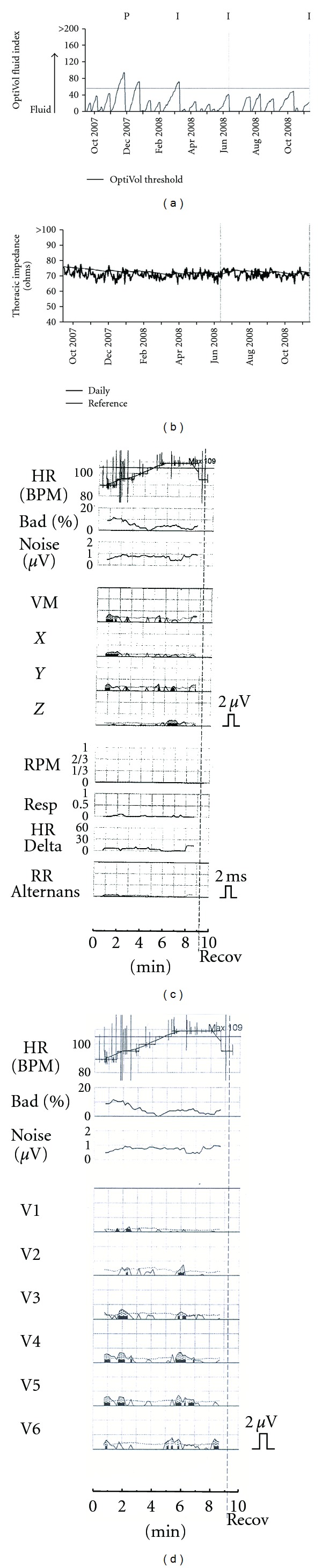
Representative data during period of clinical stability. (a) represents fluid index over time based on change in intrathoracic impedance measurements. Values beyond 60 are considered to be threshold for fluid overload. (b) represents actual intrathoracic impedance measurements over time. The solid line represents the baseline value for the patient. Lower diagrams (c and d) are T-wave alternans test data for different leads. Intrathoracic impedance values are mainly at baseline and no sustained T-wave alternans is present on a test conducted toward the end of this time period.

**Figure 2:**
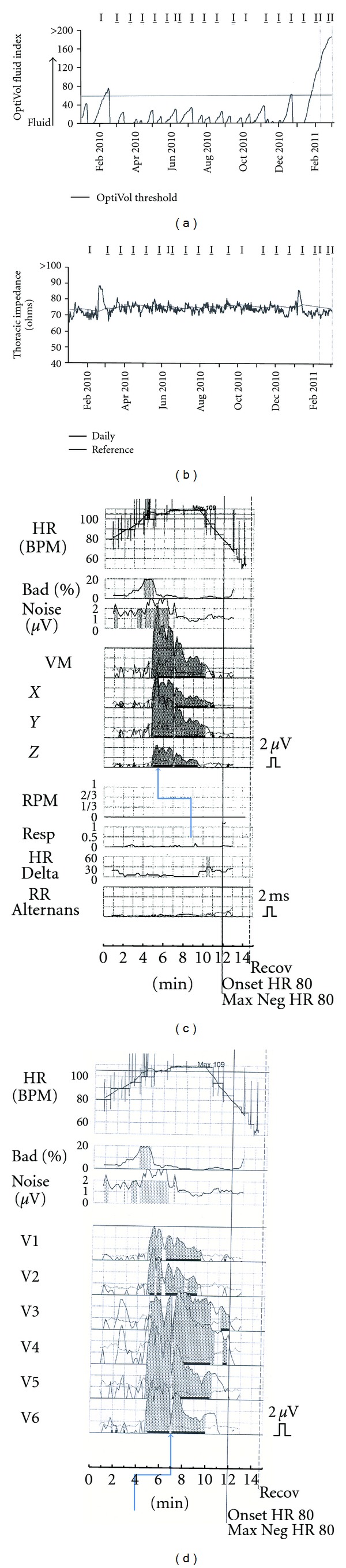
Data during heart failure exacerbation. Plots arranged as in Figure 1. The fluid index (a) is markedly elevated as intrathoracic impedance values drop (b). Sustained T-wave alternans is present as evidenced by the dark shading on the plots c and d (arrows).

**Figure 3:**
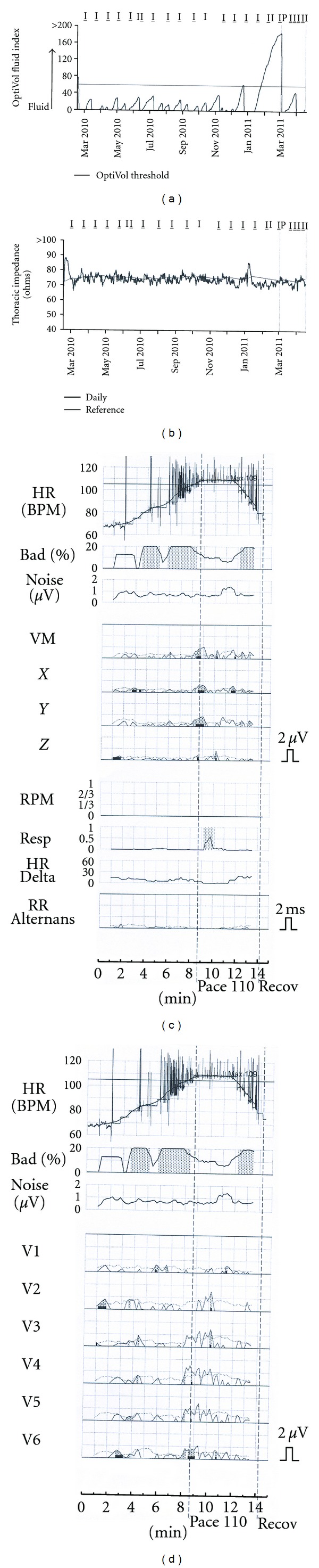
Data after patient treated for fluid overload with diuretics. The intrathoracic impedance measurements and fluid index return to baseline (a and b). No sustained alternans is present on a T-wave alternans test conducted 3 weeks after diuresis (c and d).

The current example is a 54-year-old man with nonischemic dilated cardiomyopathy and left bundle branch block. He had well-compensated heart failure following biventricular defibrillator insertion in 2007. His intrathoracic impedance measurements and T-wave alternans tests were normal at baseline (Figure 1) until March 3, 2011, when he had a heart failure exacerbation. Vital signs included blood pressure of 100/60, pulse 88, and respiratory rate 20/minute. Weight was 175 pounds, and physical exam revealed clear lungs but peripheral edema and ascites. Brain natriuretic peptide measurement (1854 pg/mL) was consistent with fluid overload, and intrathoracic impedance measurements dropped with resultant increased fluid index (Figures 2(a) and 2(b)). T-wave alternans was markedly positive with sustained alternans present at an onset heart rate of less than 105 bpm (Figures 2(c) and 2(d)). The patient was given daily doses of 80 mg intravenous furosemide until he reached his baseline weight of 168 pounds, with restoration of normal clinical status. Three weeks later, intrathoracic impedance measurements and T-wave alternans tests returned to normal (Figure 3).

## 3. Discussion

The implantable defibrillator has become standard therapy for the prevention of sudden death in patients with severe structural heart disease. The questionable cost effectiveness of routine implantation for primary prevention has resulted in the search for better methods of risk stratification. One such modality is microvolt T-wave alternans, a test that can detect beat-to-beat variations in the T-wave that may correspond to spatial dispersion of refractoriness or alternations in repolarization that underlie a substrate that can support lethal arrhythmias [6]. The utility of T-wave alternans for risk stratification has been previously investigated [1, 7].

The mechanism of T-wave alternans is unclear, but data point to abnormalities in intracellular calcium handling [2, 3, 8]. Changes in calcium handling have been shown to underlie heart failure, and conditions that lead to stretch in the heart such as volume overload are associated with altered calcium metabolism [9], arrhythmias [10], and sudden death [11]. A relationship between volume overload or stretch and T-wave alternans magnitude or susceptibility has been demonstrated in experimental models [10, 12, 13]. Thus a molecular connection in the form of altered calcium metabolism exists between heart failure, arrhythmias, and T-wave alternans. However, a correlation between T-wave alternans and cardiac volume status in the ambulatory clinical setting has not been demonstrated. 

Intrathoracic impedance measurements have been shown to correlate with cardiac fluid status and predict heart failure hospitalizations [14, 15]. Using this technology in an implantable defibrillator, we were able to detect fluid overload in a patient with cardiomyopathy, which was confirmed by physical exam and brain natriuretic peptide measurement. The dramatic increase in T-wave alternans during acute heart failure and disappearance after treatment suggest T-wave alternans status, and by extension, propensity to lethal arrhythmias, may be influenced by cardiac volume status. Such a link may help to explain the association between acute heart failure syndromes, arrhythmias, and sudden death.
